# A Combined Syndromic Approach to Examine Viral, Bacterial, and Parasitic Agents among Febrile Patients: A Pilot Study in Kilombero, Tanzania

**DOI:** 10.4269/ajtmh.17-0421

**Published:** 2017-12-26

**Authors:** Christine Hercik, Leonard Cosmas, Ondari D. Mogeni, Newton Wamola, Wanze Kohi, Eric Houpt, Jie Liu, Caroline Ochieng, Clayton Onyango, Barry Fields, Sayoki Mfinanga, Joel M. Montgomery

**Affiliations:** 1 Georgetown University, Washington, DC;; 2 Global Disease Detection Branch, Division of Global Health Protection, Center for Global Health, US Centers for Disease Control and Prevention (CDC), Nairobi, Kenya;; 3 Kenya Medical Research Institute-Centre for Global Health Research (KEMRI-CGHR), Nairobi, Kenya;; 4 National Institute of Medical Research (NIMR), Muhimbili Research Centre, Salaam, Tanzania;; 5 Division of Infectious Diseases and International Health, University of Virginia, Charlottesville, Virginia

## Abstract

The use of fever syndromic surveillance in sub-Saharan Africa is an effective approach to determine the prevalence of both malarial and nonmalarial infectious agents. We collected both blood and naso/oro-pharyngeal (NP/OP) swabs from consecutive consenting patients ≥ 1 year of age, with an axillary temperature ≥ 37.5°C, and symptom onset of ≤ 5 days. Specimens were analyzed using both acute febrile illness (AFI) and respiratory TaqMan array cards (Resp TAC) for multiagent detection of 56 different bloodstream and respiratory agents. In addition, we collected epidemiologic data to further characterize our patient population. We enrolled 205 febrile patients, including 70 children (1 < 15 years of age; 34%) and 135 adults (≥ 15 years of age; 66%). AFI TAC and Resp TAC were performed on 191 whole blood specimens and 115 NP/OP specimens, respectively. We detected nucleic acid for *Plasmodium* (57%), *Leptospira* (2%), and dengue virus (1%) among blood specimens. In addition, we detected 17 different respiratory agents, most notably, *Haemophilus influenzae* (64%), *Streptococcus pneumonia* (56%), *Moraxella catarrhalis* (39%), and respiratory syncytial virus (11%) among NP/OP specimens. Overall median cycle threshold was measured at 26.5. This study provides a proof-of-concept for the use of a multiagent diagnostic approach for exploratory research on febrile illness and underscores the utility of quantitative molecular diagnostics in complex epidemiologic settings of sub-Saharan Africa.

## INTRODUCTION

Fever is an important indicator of localized or systemic infection. The use of fever syndromic surveillance in sub-Saharan Africa is an effective approach to determine the prevalence of both malarial and nonmalarial infectious agents as well as to establish epidemiologic risk for infection. Such information is critical to provide evidence-based recommendations for local treatment approaches and disease control programming.

Recent epidemiological data suggest that because of the adoption of aggressive vector control measures, there has been a stark decline in malaria incidence across sub-Saharan Africa, particularly in Tanzania.^1–6^ In Tanzania’s South-Central region, data regarding the cause of nonmalarial febrile illness are currently lacking. At present, the malaria rapid diagnostic test is the only diagnostic tool used to direct febrile patient management in this region.^7–9^ Thus, when a patient presents with fever and tests negative for malaria, clinical care providers must rely upon symptomatic conditions alone to define provisional diagnoses and determine appropriate treatment regimens.^10–12^ Stakeholders have therefore recognized the need to improve knowledge surrounding the etiology of febrile illness, to optimize care, and to improve clinical outcomes.^3,13,14^

In the regions of sub-Saharan Africa where viral, bacterial, and parasitic infections are not well-quantified, pathogen-specific diagnostic approaches to syndromic surveillance—evaluating only one or few pathogens—are inadequate. Most of these pathogen-specific studies have solely targeted malaria, and thus failed to consider other causative agents of febrile illness.^14–24^ Given that multiple coinfections may contribute to morbidity, the detection of a single agent may not provide a complete understanding of disease etiology.

To identify the types of viral, bacterial, and parasitic organisms prevalent among febrile patients in the South-Central region of Tanzania, we conducted an exploratory investigation, surveying for 56 different microbial agents present in both blood and naso/oro-pharyngeal (NP/OP) specimens. In addition, we examined numerous clinical and epidemiologic factors among study participants.

## MATERIALS AND METHODS

### Research ethics.

This study obtained ethical clearance from the National Health Research Ethics Committee of the National Institute for Medical Research (NIMR/HQ/R.8a/Vol.IX/1735) in Tanzania.

### Study site.

**Figure 1.**
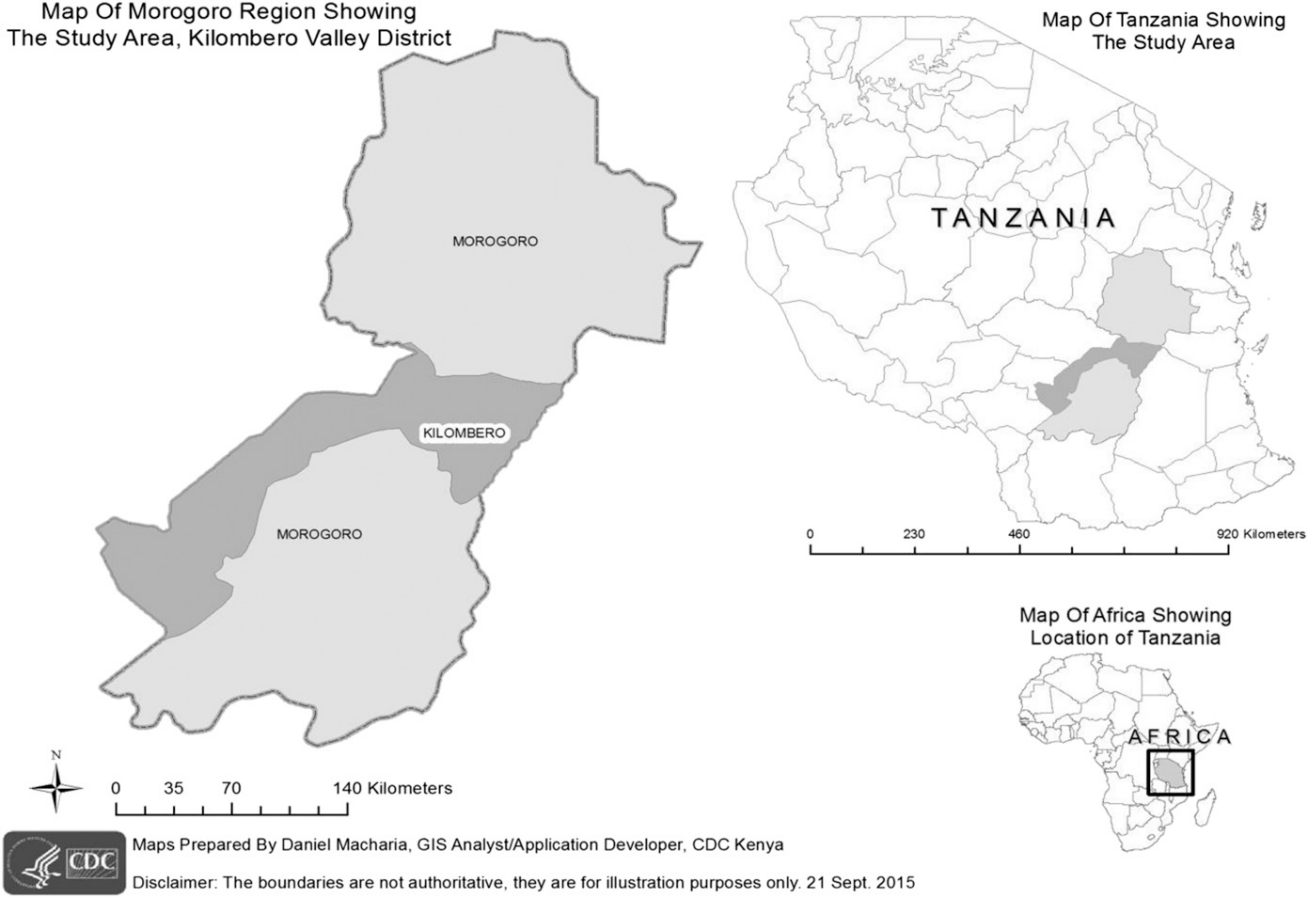
Map of study area in Kilombero district shown in dark gray in the Morogoro region inset.

This surveillance project was targeted in Tanzania’s Kilombero Valley, a rural area where agro-industrial land use associated with sugarcane production has situated a growing human population in close proximity to areas of increased wildlife biodiversity (Figure 1). The Illovo Sugar Limited Estate in Kilombero is the largest sugarcane production facility in Tanzania. The estate neighbors two national parks including: Mikumi National Park and Udzungwa Falls National Park.

This cross-sectional study was conducted at the Illovo Sugar Limited Estate Hospital (K1) and Clinic (K2). Kilombero (population > 321,000) is situated at an elevation of 300 m above mean sea level in the Morogoro Region (population > 2 million) of South-Central Tanzania. The climate is characterized by a long rainy period (March–May) and a short rainy period (October–December). Malaria transmission intensity in this region is high.^25,26^

### Study design.

The objective of this hospital-based cross-sectional study was to assess the type and prevalence of viral, bacterial, and parasitic agents detected among patients presenting with acute febrile illness (AFI) at Illovo K1 Hospital and K2 Clinic. In this regard, consenting patients with an axillary temperature ≥ 37.5°C and symptomatic onset of ≤ 5 days were eligible for enrollment. In addition to the collection of biological specimens, we collected demographic, clinical, and behavioral data to further characterize our patient population.

### Data collection.

Before enrollment, patients provided written assent for participation. Pediatric patients (1 < 15 years) also needed documented parent consent to be enrolled. Clinical, epidemiologic, and laboratory data were entered electronically onto tablets using an Open Data Kit (ODK) platform. Data were automatically submitted via the Internet to the cloud-based Formhub server.

#### Clinical assessment.

After screening and assent, a clinical officer or assistant medical officer then sought a detailed clinical history, followed by a physical examination. The attending officer entered all clinical data in a standardized electronic clinical case report form on the tablets using ODK Collect.

#### Epidemiologic evaluation.

After the clinical examination and consultation were complete, clinical officers then administered a comprehensive epidemiologic survey to further capture demographic and behavioral data. Patients under 15 years of age were administered in a pediatric epidemiologic survey, whereas patients 15 years of age and above were administered in an adult epidemiologic survey. These two survey questionnaires were similar in scope; however, they were tailored to include or omit certain age-specific inquiries (i.e., vaccination history for infants or occupational activities for adults). The wealth index was calculated using principal component analysis based on ownership of six household assets, including radio, bicycle, mobile phone, light source, television, and refrigerator.

#### Sample collection and storage.

Before initiation of antimicrobial therapy, blood was drawn from each enrolled participant. The venipuncture site was cleaned with an alcohol swab, followed by disinfection with povidone-iodine. A total of 15 mL of venous blood was extracted, including 10 mL of whole blood (in two 5 mL ethylenediaminetetraacetic acid [EDTA] tubes) for whole blood aliquotting and 5 mL of whole blood (in one 5 mL non-EDTA plain tube) for further centrifugation and aliquotting of serum. Blood samples were then stored in liquid nitrogen (−70°C) on site before specimen transport.

Subsequently, at the time of enrollment, if any adult patient was suffering from respiratory conditions (cough, difficulty breathing, chest pain, or sore throat) in addition to their febrile illness, then our laboratory technician collected both an NP/OP swab. NP/OP swabs were placed in viral transport media and stored in liquid nitrogen (−70°C) on site before specimen transport.

### Diagnostic investigations.

All blood and NP/OP specimens collected were shipped to CDC-KEMRI laboratories in Nairobi, Kenya, for further diagnostic evaluations using a syndromic TaqMan array card (TAC) diagnostic platform for the purposes of multipathogen detection of specimens. For the purposes of this study, we used both AFI and respiratory (Resp) TAC assays to screen up to 56 potential etiologic agents of illness. Serum specimens were archived in deep freezers for future analysis.

AFI TAC allows for screening of whole blood samples for the detection of 15 viruses, eight bacteria, and three protozoa in the bloodstream.^27^ Total nucleic acid was extracted from 2.5 mL of whole blood specimens using High Pure Viral Nucleic Acid Large Volume Kit. MS2 and phocine herpes virus served as built-in controls to confirm success of the extraction process and amplification efficiency. We mixed 46 µL of total nucleic acid extract with AgPath One Step RT-PCR reagents (Life Technologies, Carlsbad, CA), in a 100 µL reaction, then pipetted into the inlet port of each channel. Cards were centrifuged (1 minute, 1,200 rpm twice), sealed, and the inlet ports removed as directed by the manufacturer’s instructions. All AFI TACs were run on the ViiA^™^ seven real-time PCR system (Life Technologies) using PCR cycling conditions comprising 10 minutes at 50°C, 20 seconds at 95°C, followed by 45 two-step cycles of 3 seconds at 95°C and 30 seconds at 60°C.^27^

Resp TAC further allows for screening of NP/OP specimens for the detection of 15 viruses and 15 bacteria known to cause respiratory illness. Total nucleic acid was extracted from 100 µL of NP/OP in MagNA Pure 96 Instrument (Roche) using MagNA Pure 96 DNA and Viral NA Small Volume Kit (Roche, Indianapolis, IN) and eluted in 100 μL of elution buffer. Forty-six microliters of total nucleic acid extract was mixed with AgPath One Step RT-PCR reagents (Life Technologies), in a 100 µL reaction, then pipetted into the inlet port of each channel. Cards were centrifuged (1 minute, 1,200 rpm twice), sealed, and the inlet ports removed as directed by the manufacturer’s instructions. Resp TACs were run on the ViiA seven real-time PCR system (Life Technologies) using PCR cycling conditions comprising 45°C for 10 minutes, 94°C for 10 minutes, and 45 cycles of 94°C for 30 seconds followed by 60°C for 1 minute.^28^

Our team captured additional laboratory testing performed by clinical staff as part of routine patient care, including malaria rapid diagnostic tests and microscopy. In this regard, clinicians administered an SD Bioline malaria Ag P.f/Pan rapid diagnostic test (Alere Inc., Waltham, MA) at point-of-care for all participants. If the patient yielded a positive rapid test result, a microscopic examination of Giemsa-stained thick blood film smears was performed to determine the intensity of infection. Results were scored as 0 (no parasites), 1+ (1–9 parasites/µL), 2+ (10–19 parasites/µL), 3+ (20–29 parasites/µL), or 4+ (30 or more parasites/µL).

### Statistical analysis.

Statistical analyses were performed using SAS version 9.3 software (SAS Inc., Cary, NC). Descriptive statistics was presented as medians, ranges, and interquartile ranges for continuous variables and as proportions for categorical variables.

To better interpret which agents may in fact be contributing to fever syndrome, we further investigated cycle threshold (Ct) values, which serve as proxies for microbial concentration. We examined distributions of Ct values by agent using box plots and, based upon these distributions, we calculated the overall median Ct observed among all detected agents, for comparison to agent-specific Ct median values.

Furthermore, to explore potential correlation between parasite load (Ct), as determined by quantitative PCR (qPCR), and the intensity of infection, as determined by blood smear, we conducted an analysis of variance (ANOVA) test to examine mean Ct values for patients grouped within each level of parasite intensity (1+, 2+, 3+, or 4+).

## RESULTS

### Patient screening and enrollment.

From June 11 to July 12, 2014, a total of 250 patients were screened to determine their eligibility for enrollment. Of these patients, 205 febrile patients were enrolled, including 70 children (1 < 15 years of age; 34%) and 135 adults (≥ 15 years of age; 66%). All patients enrolled were Tanzanian, and 151 (73.7%) lived on the grounds of the Estate.

Male enrollment (66%) was more common than female enrollment. The median age of enrollment was 23 years (range 1–80 years) (Table 1). The most common presenting complaints, other than fever, were headache (78%), lethargy (42%), cough (34%), and vomiting (29%). Of all enrolled participants, 116 (57%) were admitted for inpatient care and treatment (Table 2). Patient-level data, inclusive of particular clinical and epidemiologic data as well as diagnostic results, are included in a supplemental file (Supplemental Table 1: Clinical, epidemiologic and diagnostic findings from enrolled participants).

**Table 1:** Demographic and socioeconomic characteristics of enrolled febrile pediatric and adult patients

	Pediatric (1–14 years) (*N* = 70)	Adult (15+ years) (*N* = 135)	Total (*N* = 205)
Indicator	*n* (%)	*n* (%)	*n* (%)
Gender			
Female	28 (40.0%)	42 (31.1%)	70 (34.1%)
Age (years)			
Median (IQR)	5.3 (2.7–8.8)	29.8 (23.2–38.2)	23.0 (8.5–33.3)
Mean (SD)	6.1 (3.9)	32.4 (12.8)	23.4 (16.4)
Residence			
Live on estate grounds	44 (62.9%)	107 (79.3%)	151 (73.7%)
Live outside estate grounds	26 (37.1%)	28 (20.7%)	54 (26.3%)
Education level			
Under age for formal education	34 (48.6%)	0 (0%)	34 (16.6%)
No formal education	14 (20%)	5 (3.7%)	53 (25.9%)
Incomplete primary school	20 (28.6%)	10 (7.4%)	30 (14.6%)
Completed primary school	1 (1.4%)	79 (58.5%)	80 (39%)
Incomplete secondary school	0 (0%)	6 (4.4%)	6 (2.9%)
Completed secondary school	0 (0%)	32 (23.7%)	32 (15.6%)
Completed vocational school and/or university	0 (0%)	3 (2.2%)	3 (1.5%)
Residence status			
Full time	69 (98.6%)	101 (74.8%)	170 (82.9%)
Part time (seasonal)	1 (1.4%)	33 (24.4%)	34 (16.6%)
Part time (weekends)	0 (0%)	1 (0.7%)	1 (0.5%)
Occupation at Illovo estate			
Sugarcane cutting	0 (0%)	40 (29.6%)	40 (19.5%)
Weeding	0 (0%)	17 (12.6%)	17 (8.3%)
Factory work	0 (0%)	14 (10.4%)	14 (6.8%)
Managerial work	0 (0%)	3 (2.2%)	3 (1.5%)
Security work	0 (0%)	1 (0.7%)	1 (0.5%)
Other estate work	0 (0%)	13 (9.6%)	13 (6.3%)
Not used by the estate	0 (0%)	46 (34.1%)	46 (22.4%)
Wealth quintile (based on household possessions)			
1 (poorest)	8 (11.4%)	39 (28.9%)	47 (22.9%)
2	21 (30%)	35 (25.9%)	56 (27.3%)
3	5 (7.1%)	16 (11.9%)	21 (10.2%)
4	17 (24.3%)	23 (17%)	40 (19.5%)
5 (wealthiest)	19 (27.1%)	22 (16.3%)	41 (20%)

IQR = interquartile range; SD = standard deviation.

**Table 2:** Clinical characteristics of enrolled febrile pediatric and adult patients

	Pediatric (1–14 years) (*N* = 70)	Adult (15+ years) (*N* = 135)	Total (*N* = 205)
Indicator	*n* (%)	*n* (%)	*n* (%)
Axillary temperature (°C)			
37.5–38.4	40 (57.1%)	77 (57%)	117 (57.1%)
38.5–39.4	19 (27.1%)	41 (30.4%)	60 (29.3%)
39.5–40.4	10 (14.3%)	17 (12.6%)	27 (13.2%)
40.5+	1 (1.4%)	0 (0%)	1 (0.5%)
Mean (SD)	38.5 (0.8)	38.5 (0.8)	38.5 (0.8)
Median (IQR)	38.2 (37.8–38.2)	38.3 (37.9–38.3)	38.3 (37.8–38.3)
Recent weight loss			
Yes	15 (21.4%)	19 (14.1%)	34 (16.6%)
Chief complaints			
Headache	46 (65.7%)	113 (83.7%)	159 (77.6%)
Lethargy	29 (41.4%)	57 (42.2%)	86 (42%)
Cough	30 (42.9%)	40 (29.6%)	70 (34.1%)
Vomiting	26 (37.1%)	34 (25.2%)	60 (29.3%)
Abdominal pain	23 (32.9%)	25 (18.5%)	48 (23.4%)
Chest pain	5 (7.1%)	28 (20.7%)	33 (16.1%)
Diarrhea	9 (12.9%)	18 (13.3%)	27 (13.2%)
Sore throat	3 (4.3%)	12 (8.9%)	15 (7.3%)
Pain when urinating	1 (1.4%)	12 (8.9%)	13 (6.3%)
Ear pain	1 (1.4%)	4 (3%)	5 (2.4%)
Rash or red eyes	1 (1.4%)	0 (0%)	1 (0.5%)
Presentation with respiratory illness			
Yes	31 (44.3%)	53 (39.3%)	84 (41%)
HIV status (self-reported)			
Positive	1 (1.4%)	2 (1.5%)	3 (1.5%)
Negative	13 (18.6%)	42 (31.1%)	55 (26.8%)
Unknown	56 (80%)	91 (67.4%)	147 (71.7%)
Admission status			
Admitted	41 (58.6%)	75 (55.6%)	116 (56.6%)

IQR = interquartile range; SD = standard deviation.

### Laboratory results.

Diagnostic evaluations using both AFI and Resp TAC identified 20 different viral, bacterial, and parasitic agents among 191 febrile patients contributing blood specimens and 115 febrile patients contributing NP/OP specimens. We did not detect nucleic acid for any of the 56 surveyed agents in 38 (20%) febrile participants (Table 3).

**Table 3:** The proportion of enrolled pediatric and adult febrile patients with detected nucleic acid for examined viral, bacterial and parasitic agents on acute febrile illness and Respiratory TaqMan array cards

	Pediatric			Adult			All		
Agent identified	N positive	N tested	% Positive	N positive	N tested	% Positive	N positive	N tested	% Positive
Bloodstream detections									
*Plasmodium*	31	58	53	77	133	58	108	191	57
*Leptospira*	0	58	0	3	133	2	3	191	2
Dengue virus	0	58	0	1	133	1	1	191	1
Naso/Oro-pharyngeal detections									
Adenovirus	10	51	20	5	64	8	15	115	13
Enterovirus	7	51	14	1	64	2	8	115	7
Influenza B	2	51	4	2	64	3	4	115	4
*Group A Streptococcus*	11	51	22	8	64	13	19	115	17
Coronavirus 229E	5	51	10	3	64	5	8	115	7
Coronavirus NL63	0	51	0	1	64	2	1	115	1
*Haemophilus influenzae-*pan	42	51	82	32	64	50	74	115	64
*H. influenzae-*type B_1	3	51	6	1	64	2	4	115	4
*H. influenzae–*type B_2	0	51	0	1	64	2	1	115	1
*Klebsiella pneumoniae*	0	51	0	2	64	3	2	115	2
*Moraxella catarrhalis*	37	51	73	8	64	13	45	115	39
Parainfluenza virus 1	1	51	2	0	64	0	1	115	1
*Pseudomonas aeruginosa*	4	51	8	4	64	6	8	115	7
Respiratory syncytial virus	7	51	14	5	64	8	12	115	11
Human rhinovirus	9	51	18	5	64	8	14	115	12
*Staphylococcus aureus*	15	51	29	12	64	19	27	115	24
*Streptococcus pneumoniae*	35	51	70	29	64	45	64	115	56

#### Bloodstream detections.

AFI TAC was performed on 191 febrile patients. Of these, we detected nucleic acid for *Plasmodium* (108; 57%), *Leptospira* (3; 2%), and dengue virus (1; 1%). [Note: as part of quality assurance, the single positive dengue result by AFI TAC (sample ID: 204113015) was confirmed by individual reverse transcription PCR.] Overall, we detected nucleic acid for bloodstream agents in 31 (53%) children and 81 (61%) adults. *Plasmodium* was found to be the most common agent detected (57%), with a fairly even distribution between pediatric (53%) and adult (58%) participants.

#### NP/OP detections

In addition, Resp TAC was performed on 115 febrile cases suspected of respiratory infection. Of these, we detected nucleic acid for 17 different organisms, most notably: *Haemophilus influenzae* (74; 64%), *Streptococcus pneumoniae* (64; 56%), *Moraxella catarrhalis* (45; 39%), and *Staphylococcus aureus* (27; 24%). The more common upper respiratory agents surveyed tended to be more prevalent among pediatric patients as compared with adult participants, including adenovirus (pediatrics: 20%; adults: 8%), enterovirus (pediatrics: 14%; adults: 2%), *group A Streptococcus* (pediatrics: 22%; adults: 13%), and human rhinovirus (pediatrics: 18%; adults: 8%). While we did not detect evidence of influenza A, we did detect influenza B among four individuals (pediatrics: 4%; adults: 3%).

#### Multiple codetections.

**Figure 2.**
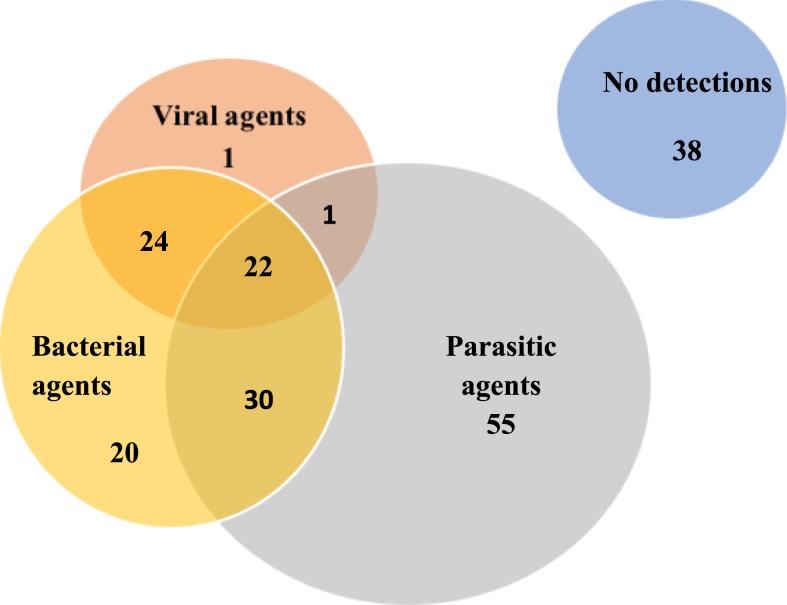
Overlap among agent types, indicating single and multiple type codetections among enrolled febrile patients. Note: Figure 2 is not to scale. This figure appears in color at www.ajtmh.org.

We detected nucleic acid for multiple organisms in a significant proportion of enrolled individuals (96; 50%), with detection of at least four or more organisms in about a quarter of all febrile participants (45; 24%) (Table 4). We concomitantly detected nucleic acid for bacterial and parasitic agents among 30 participants (16%), bacterial and viral agents among 24 participants (13%), and viral and parasitic agents in one (0.5%) individual (Figure 2).

**Table 4:** Frequency of detection of single and multiple organisms

Detection frequency	*N*	%
No organism detected	38	19.9
One organism detected	57	29.8
Codetection of two organisms	24	12.6
Codetection of three organisms	27	14.1
Codetection of four organisms	23	12.0
Codetection of five organisms	12	6.3
Codetection of six organisms	8	4.2
Codetection of seven organisms	2	1.0

**Figure 3.**
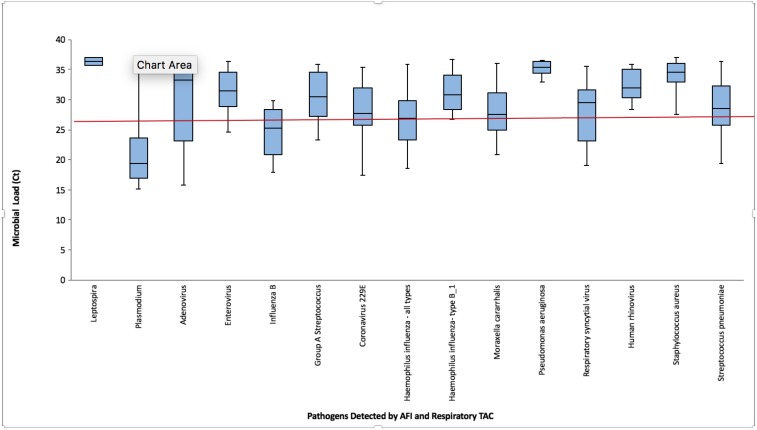
The box plot of cycle threshold (Ct) distributions of detected organisms among febrile patients, with the overall median Ct value (26.5) denoted by the red line. This figure appears in color at www.ajtmh.org.

To better interpret our quantitative diagnostic results, we examined Ct distributions among all agents detected. Figure 3 depicts Ct distributions, as observed by agent, with overall median Ct measured at 26.5. Ct medians of both *Plasmodium* and influenza B were detected at 18.5 and 25 respectively, thus indicating higher concentrations as compared with other detected organisms.

**Figure 4.**
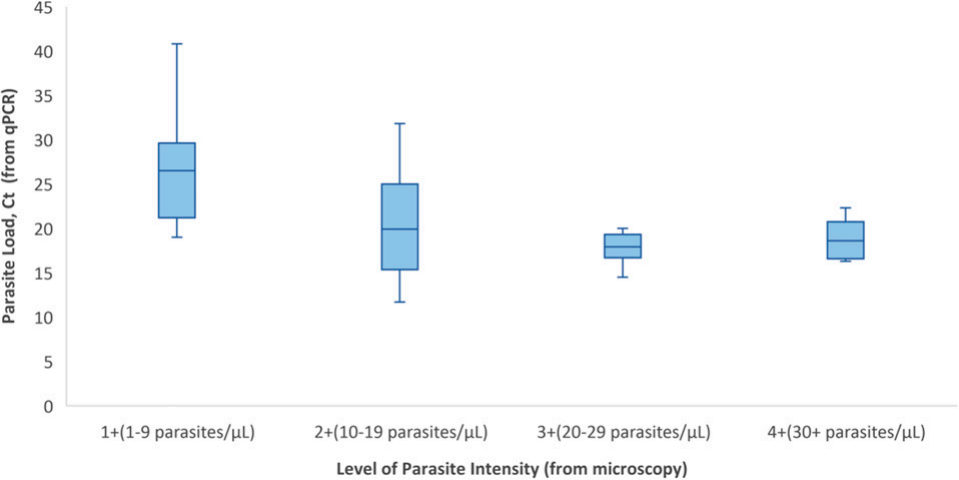
Box plot of parasite load (Ct) by the level of parasite intensity. This figure appears in color at www.ajtmh.org.

In addition, for those patients in which we had a microscopy result, we conducted an ANOVA test to examine mean Ct values, as determined by qPCR, for patients grouped within each level of parasite intensity (1+, 2+, 3+, or 4+), as determined by blood smear. Because low Ct values correspond to high parasite loads, we would expect that lower mean Ct values correlate with higher levels of parasite intensity. Results were statistically significant (*P* value = 0.0025) and findings demonstrate an inverse relationship between mean Ct values and level of parasite intensity. As depicted in Figure 4, we observe lower mean Ct values (i.e., higher detected numbers of *Plasmodium* parasites) correlated with higher levels of parasite intensity.

## DISCUSSION

The lack of consistency in methodological approaches to AFI surveillance in low-resource areas has resulted in an inability to provide more comprehensive information on a wide range of organisms potentiating fever syndrome.^10,14,29–31^ Our study seeks to provide a platform for preliminary exploratory research to determine the range of biological agents to consider during clinical assessment of fever syndrome. This is the first field study to investigate viral, bacterial, and parasitic agents of febrile illness using the syndromic AFI TAC diagnostic platform. The TAC system allows researchers to screen each specimen for a suite of microbial agents, rather than conduct multiple rounds of testing using pathogen-specific detection methods.

Our study eligibility criteria and case definitions were not optimized to detect any specific pathogen. We maintained broad and inclusive enrollment criteria given the exploratory nature of our investigation. Of the 56 agents surveyed, *Plasmodium* was found to be the most common microbe detected, which was consistent with our a priori hypothesis. Given that Kilombero is located in a highly endemic area for malaria, the detection of *Plasmodium* parasitemia in older children and adults is not unexpected and is not necessarily indicative of fever etiology. However, as observed in overall Ct distributions, *Plasmodium* was detected with the lowest median Ct value (18.5), signaling relatively high parasite loads among affected individuals. It is also important to recognize that our pilot survey was conducted during the month of June, at the peak season of malaria transmission.

In addition to the detection of *Plasmodium* in the bloodstream, we also detected dengue virus and *Leptospira*. Dengue virus was detected in one individual, with no reported recent travel history or residence status elsewhere, implying that this case was a locally acquired infection. Dengue virus has been reported in the coastal regions of Tanzania and has recently been detected as far inland as the Kilosa District.^32,33^ To our knowledge, this is the first study to detect dengue virus in Kilombero; therefore suggesting continued geographic spread of the virus. *Leptospira* has been previously investigated in the Northern areas of Tanzania, and research has suggested potential endemicity in the Kilimanjaro region.^34^ Further research is needed to determine the prevalence of *Leptospira* in the Kilombero area as well as to determine potential reservoirs and risk factors associated with infection.

In addition to bloodstream pathogens, we detected a range of bacterial and viral agents present in NP/OP specimens from febrile patients. While we did not detect influenza A, we did detect four cases of influenza B. These results are consistent with findings from a recent cross-sectional study in the Kilosa District, whereby the prevalence of influenza B was higher as compared with influenza A.^32^ These results differ, however, from a national-level influenza survey, whereby researchers detected a significantly higher prevalence of influenza A (7%) as compared with influenza B (1%).^35^

Previous literature has suggested an association between lower Ct values and manifestation of a more severe clinical illness.^36–39^ For cases presenting with influenza B (4; 4%) and *Plasmodium* (108; 57%), we detected lower-than-average median Ct values, indicating relatively high microbial loads and further hypothesize that these agents are more likely to be contributing to disease status. We recognize that it is important to consider additional biomarkers, beyond the concentration of microbial load, to better elucidate etiology. Further study is needed to examine patient immune response, such as cytokine or other inflammatory markers as well as antibody titers, to better support etiologic determination.

We detected multiple agents in a significant proportion of febrile participants (96; 50%). Of those agents commonly codetected, we detected *Plasmodium*, *H. influenzae*, and *M. catarrhalis* as the most frequent combination among febrile patients (9; 7.8%). These findings are consistent with a recent study in understanding the etiology of AFI among children in the northeastern part of Tanzania, whereby coinfections were observed in about a quarter of all febrile patients.^40^ The coexistence of multiple infectious organisms can serve as an obstacle in developing fever management guidelines, particularly in poor rural areas of sub-Saharan Africa where patients tend to suffer from numerous kinds of concurrent infections.^40^ While this study provided a preliminary landscape of the range of biological agents copresenting among febrile patients, a deeper understanding of both the prevalence as well as clinical ramifications of this circumstance will be critical for developing appropriate care and treatment guidelines for these individuals.

Our study had several limitations. First, while we wanted to combine two TAC assays to enhance broad-spectrum investigation of febrile illness, each TAC assay requires a different type of clinical specimen, thus comparing diagnostic outputs can be difficult. While organisms detected in whole blood indicate viremia, bactaeremia, and/or parasitemia, organisms detected from the pharynges may only be representative of microbial carriage. For the purposes of this study, we did not state final determination of disease etiology, but we included agents in our analyses if nucleic acid was detected and presented distributions of Ct for relative quantification of microbial loads. In addition, while AFI and Resp TAC are both validated diagnostic techniques, the specificity and sensitivity of these assays are not 100%.^27,28^

In addition, this pilot survey was limited both temporally and geographically and yielded a rather small sample size of 205 participants. Given the varying seasonal patterns of both respiratory, as well as vector-borne diseases, different results could have been obtained if this study had been conducted in another region or during another season or year. Furthermore, given resource and testing constraints of this study, we solely enrolled febrile patients meeting our enrollment criteria, and thus did not enroll community- or hospital-based controls for subsequent analyses.

In light of these limitations, this study provided an initial, exploratory examination of the various viral, bacterial, and parasitic agents prevalent among febrile patients in our study area and offered a highly sensitive diagnostic methodology for broad-spectrum consideration of disease etiology. This pilot survey provided a baseline for the scale-up of a subsequent yearlong surveillance study to further explore seasonality of illness over a longer time period, with a more robust sample size. The subsequent longitudinal study will further allow our study team to conduct needed epidemiologic analysis to evaluate factors associated with infection status.

## Supplementary Material

Supplemental Table.
