# Supplementary material for: A Combined Syndromic Approach to Examine Viral, Bacterial, and Parasitic Agents among Febrile Patients: A Pilot Study in Kilombero, Tanzania
Source: Am J Trop Med Hyg. 2017 Dec 26;98(2):625–32. doi: 10.4269/ajtmh.17-0421 (PMC5929188; doi:10.4269/ajtmh.17-0421)
Supplement: Supplementary file 1 [file tpmd170421.SD1.pdf]

Supplemental Table 1

Clinical, epidemiologic, and diagnostic findings from enrolled participants

Sample ID	Gender	Age	Axillary temperature (°C)	Admission	Malaria RDT completed?	RDT result	Microscopy completed?	Microscopy result	AFI TAC completed?	Dectection(s) by AFI TAC result	Respiratory TAC completed?	Detection(s) by respiratory TAC
2014112964	Male	33.55	37.5	No	Yes	POS	Yes	4+	Yes	None	Yes	None
2014112968	Female	24.04	37.5	No	Yes	POS	Yes	2+	Yes	*Plasmodium*	Yes	None
2014112969	Female	33.95	37.5	Yes	Yes	NEG	No	–	Yes	*Plasmodium*	Yes	None
2014112971	Female	80	37.5	No	Yes	NEG	No	–	Yes	None	Yes	None
2014112972	Male	23.78	37.8	Yes	Yes	POS	Yes	2+	Yes	None	Yes	None
2014112975	Male	32.03	38	Yes	Yes	POS	Yes	4+	Yes	*Plasmodium*	Yes	None
2014112976	Male	56.92	39.2	Yes	Yes	POS	Yes	4+	Yes	*Plasmodium*	Yes	None
2014112981	Male	24.66	39	Yes	Yes	NEG	No	–	Yes	*Plasmodium*	Yes	None
2014113004	Male	34.84	39.8	No	Yes	NEG	No	–	Yes	*Plasmodium*	Yes	None
2014113006	Female	53.64	39	Yes	Yes	NEG	No	–	Yes	*Plasmodium*	Yes	None
2014113012	Female	41.11	37.8	Yes	Yes	NEG	No	–	Yes	None	Yes	None
2014113069	Male	36.01	38	Yes	Yes	POS	Yes	3+	Yes	None	Yes	None
2014113072	Female	6.091	38.6	Yes	Yes	NEG	Yes	No parasite	Yes	*Plasmodium*	Yes	*Haemophilus* influenza-all types
2014113073	Male	24.55	38.6	Yes	Yes	NEG	Yes	No parasite	Yes	*Plasmodium*	Yes	*Streptococcus pneumoniae*
2014113074	Male	15.85	38.4	Yes	Yes	POS	Yes	No parasite	Yes	*Plasmodium*	Yes	*Haemophilus* influenza-all types
2014113075	Male	22.03	40	No	Yes	POS	Yes	No parasite	Yes	*Plasmodium*	Yes	*Haemophilus* influenza-all types
2014113076	Male	61.34	37.6	Yes	Yes	NEG	No	–	Yes	Dengue	Yes	*Staphylococcus aureus*
2014113077	Male	22.39	39.8	Yes	Yes	POS	Yes	No parasite	Yes	*Plasmodium*	Yes	*S. pneumoniae*
2014113078	Female	45.53	37.9	Yes	Yes	POS	Yes	2+	Yes	None	Yes	*S. pneumoniae*
2014113079	Female	38.86	39.3	Yes	Yes	POS	Yes	No parasite	Yes	*Plasmodium*	Yes	*Moraxella catarrhalis*
2014113080	Female	22.52	37.5	Yes	Yes	POS	No	–	Yes	*Plasmodium*	Yes	*S. pneumoniae*
2014113081	Female	19.02	40.3	No	Yes	POS	Yes	No parasite	Yes	Lepto	Yes	*S. pneumoniae*
2014113082	Male	26.17	39.3	No	Yes	NEG	Yes	No parasite	Yes	*Plasmodium*	Yes	*Haemophilus* influenza-all types
2014113084	Male	29.5	38	Yes	Yes	POS	Yes	3+	Yes	*Plasmodium*	Yes	Adenovirus
2014113085	Female	31.02	37.7	Yes	Yes	POS	Yes	2+	Yes	*Plasmodium*	Yes	*S. aureus*
2014113086	Male	20.5	38	Yes	Yes	POS	Yes	3+	Yes	*Plasmodium*	Yes	*S. pneumoniae*
2014113087	Female	20.05	37.8	Yes	Yes	POS	Yes	4+	Yes	*Plasmodium* + lepto	Yes	Human rhinovirus
2014113088	Male	35.07	38	Yes	Yes	NEG	Yes	No parasite	Yes	*Plasmodium*	Yes	*S. aureus*
2014113089	Male	38.18	37.6	No	Yes	NEG	No	–	Yes	*Plasmodium*	Yes	*Haemophilus* influenza-all types + *S. pneumoniae*
2014113090	Male	36.3	38.8	No	Yes	NEG	No	–	Yes	None	Yes	Group A *Streptococcus* + *S. aureus*
2014113091	Male	3.712	37.7	No	Yes	NEG	No	–	No	None	Yes	*Haemophilus* influenza-all types + *S. pneumoniae*
2014113092	Female	4.054	39.5	Yes	Yes	NEG	Yes	No parasite	Yes	*Plasmodium*	Yes	*Haemophilus* influenza-all types + *S. pneumoniae*
2014113093	Male	27.59	39	Yes	Yes	POS	Yes	4+	Yes	None	Yes	Enterovirus + *M. catarrhalis*
2014113094	Male	1.848	37.7	Yes	Yes	NEG	No	–	Yes	None	Yes	Coronavirus 229E + respiratory syncytial virus
2014113095	Male	15.3	38.6	No	Yes	POS	No	No parasite	Yes	*Plasmodium*	Yes	*Haemophilus* influenza-all types + *S. aureus*
2014113097	Male	22.31	38.4	Yes	Yes	POS	Yes	No parasite	Yes	*Plasmodium*	Yes	*Haemophilus* influenza-all types + *S. pneumoniae*
2014113098	Male	23.67	37.6	No	Yes	NEG	No	–	Yes	None	Yes	*Haemophilus* influenza-all types + *S. pneumoniae*
2014113099	Male	10.53	38.2	No	Yes	POS	Yes	No parasite	Yes	None	Yes	Group A *Streptococcus* + *S. aureus*
2014113100	Female	43.04	37.5	No	Yes	NEG	No	–	Yes	None	Yes	*Pseudomonas aeruginosa* + respiratory syncytial virus
2014113101	Male	10.78	38.5	Yes	Yes	POS	Yes	No parasite	Yes	*Plasmodium*	Yes	*Haemophilus* influenza-all types + *S. aureus*

(continued)

Supplemental Table 1

Continued

Sample ID	Gender	Age	Axillary temperature (°C)	Admission	Malaria RDT completed?	RDT result	Microscopy completed?	Microscopy result	AFI TAC completed?	Dectection(s) by AFI TAC result	Respiratory TAC completed?	Detection(s) by respiratory TAC
2014113102	Male	31.46	40	Yes	Yes	POS	Yes	No parasite	Yes	*Plasmodium*	Yes	*Haemophilus* influenza-all types + *S. pneumoniae*
2014113103	Male	21.21	38.6	No	Yes	NEG	No	–	Yes	*Plasmodium*	Yes	*Haemophilus* influenza-all types + *S. pneumoniae*
2014113105	Male	32.28	38	Yes	Yes	POS	Yes	1+	Yes	*Plasmodium*	Yes	Coronavirus 229E + *Haemophilus* influenza-all types
2014113108	Male	25.13	39.7	No	Yes	NEG	No	–	Yes	None	Yes	*Haemophilus* influenza-all types + *S. pneumoniae*
2014113109	Male	10.49	39.7	Yes	Yes	POS	Yes	No parasite	Yes	*Plasmodium*	Yes	*Haemophilus* influenza-all types + *M. catarrhalis*
2014113110	Male	22.03	39.3	Yes	Yes	POS	No	–	Yes	None	Yes	*Haemophilus* influenza-all types + human rhinovirus
2014113111	Male	24.03	37.5	Yes	Yes	NEG	No	–	Yes	*Plasmodium*	Yes	Human rhinovirus + *S. pneumoniae*
2014113112	Male	22.02	38	Yes	Yes	NEG	No	–	Yes	*Plasmodium*	Yes	*Haemophilus* influenza-all types + *S. pneumoniae*
2014113113	Male	27.68	38.8	No	Yes	POS	Yes	1+	Yes	*Plasmodium*	Yes	Influenza B + *Haemophilus* influenza-all types
2014113114	Male	12.57	39.4	Yes	Yes	POS	Yes	1+	Yes	*Plasmodium*	Yes	*S. aureus* + *S. pneumoniae*
2014113115	Male	24.15	38	Yes	Yes	POS	Yes	2+	No	None	Yes	*Haemophilus* influenza-type B2 + *Klebsiella pneumoniae*
2014113116	Male	3.646	38.8	Yes	Yes	NEG	Yes	No parasite	No	None	Yes	*Haemophilus* influenza-all types + *S. pneumoniae*
2014113117	Male	45.02	37.8	Yes	Yes	POS	Yes	1+	Yes	None	Yes	*S. aureus* Bc + *S. pneumoniae*
2014113118	Male	14.2	38.3	No	Yes	POS	Yes	2+	Yes	None	Yes	Group A *Streptococcus* + Psae + *S. aureus*
2014113119	Female	36	40	Yes	Yes	POS	Yes	No parasite	Yes	None	Yes	Adenovirus + respiratory syncytial virus + *S. pneumoniae*
2014113121	Male	39.06	38.2	No	Yes	NEG	Yes	No parasite	Yes	*Plasmodium*	Yes	*Haemophilus* influenza-all types + respiratory syncytial virus + *S. pneumoniae*
2014113122	Male	26.26	38.8	No	Yes	NEG	Yes	No parasite	Yes	*Plasmodium*	Yes	Group A *Streptococcus* + *M. catarrhalis* + *S. pneumoniae*
2014113123	Female	6.967	38.3	No	Yes	NEG	Yes	No parasite	Yes	None	Yes	*Haemophilus* influenza-all types + *M. catarrhalis* + *S. pneumoniae*
2014113124	Female	5.913	37.6	No	Yes	POS	No	–	Yes	None	Yes	*Haemophilus* influenza-all types + *M. catarrhalis* + *S. pneumoniae*
2014113125	Male	12.27	40.7	Yes	Yes	POS	Yes	No parasite	Yes	*Plasmodium*	Yes	Adenovirus + enterovirus + *Haemophilus* influenza-all types
2014113126	Male	3.707	38	No	Yes	POS	Yes	2+	Yes	*Plasmodium*	Yes	*Haemophilus* influenza-all types + *M. catarrhalis* + *S. pneumoniae*
2014113128	Male	24.48	39	Yes	Yes	NEG	Yes	No parasite	Yes	*Plasmodium*	Yes	*Haemophilus* influenza-all types + *S. aureus* + *S. pneumoniae*
2014113129	Female	12.85	38.1	Yes	Yes	POS	Yes	3+	Yes	None	Yes	*M. catarrhalis* + respiratory syncytial virus + *S. pneumoniae*
2014113130	Male	14.57	38.8	No	Yes	POS	Yes	2+	Yes	None	Yes	Group A *Streptococcus* + *Haemophilus* influenza-all types + *M. catarrhalis*
2014113131	Male	6.069	37.5	No	Yes	POS	Yes	4+	Yes	None	Yes	*Haemophilus* influenza-all types + *M. catarrhalis* + *S. pneumoniae*

(continued)

Supplemental Table 1
Continued

Sample ID	Gender	Age	Axillary temperature (°C)	Admission	Malaria RDT completed?	RDT result	Microscopy completed?	Microscopy result	AFI TAC completed?	Dectection(s) by AFI TAC result	Respiratory TAC completed?	Detection(s) by respiratory TAC
2014113133	Male	47	39.4	Yes	Yes	NEG	Yes	No parasite	Yes	*Plasmodium*	Yes	*Haemophilus* influenza-all types + *P. aeruginosa* + *S. pneumoniae*
2014113134	Male	33.28	39.3	No	Yes	POS	Yes	2+	Yes	*Plasmodium*	Yes	Group A *Streptococcus* + *Haemophilus* influenza-all types + *S. aureus*
2014113135	Male	19.4	38.3	Yes	Yes	NEG	No	–	Yes	None	Yes	Influenza B + *Haemophilus* influenza-all types + *S. pneumoniae*
2014113136	Female	3.874	37.5	No	Yes	POS	Yes	2+	Yes	None	Yes	Adenovirus + *Haemophilus* influenza-all types + *M. catarrhalis*
2014113137	Female	5.42	38.1	No	Yes	NEG	No	–	Yes	None	Yes	Influenza B + *M. catarrhalis* + *S. pneumoniae*
2014113138	Female	21.02	38	Yes	Yes	POS	Yes	No parasite	Yes	*Plasmodium*	Yes	*Haemophilus* influenza-all types + *M. catarrhalis* + human rhinovirus
2014113139	Female	31.35	38	No	Yes	NEG	No	–	Yes	*Plasmodium*	Yes	*Haemophilus* influenza-all types + *M. catarrhalis* + *S. pneumoniae*
2014113140	Female	2.346	38.7	Yes	Yes	POS	Yes	3+	Yes	None	Yes	*Haemophilus* influenza-all types + *M. catarrhalis* + *S. pneumoniae*
2014113142	Female	16.15	37.5	No	Yes	POS	Yes	No parasite	Yes	None	Yes	Group A *Streptococcus* + *Haemophilus* influenza-all types + human rhinovirus
2014113143	Male	1.826	39	Yes	Yes	POS	Yes	No parasite	No	None	Yes	Adenovirus + *Haemophilus* influenza-all types + *M. catarrhalis*
2014113144	Male	2.08	37.5	No	Yes	POS	Yes	No parasite	Yes	None	Yes	Human rhinovirus + *S. aureus* + *S. pneumoniae*
2014113145	Female	79.01	39.1	Yes	Yes	NEG	No	–	Yes	*Plasmodium*	Yes	*Haemophilus* influenza-all types + *P. aeruginosa* + respiratory syncytial virus
2014113146	Male	31.37	37.8	No	Yes	POS	Yes	2+	Yes	*Plasmodium*	Yes	Group A *Streptococcus* + *Haemophilus* influenza-all types + *M. catarrhalis* + *S. pneumoniae*
2014113147	Male	1.702	38.2	No	Yes	POS	Yes	No parasite	Yes	None	Yes	Enterovirus + Coronavirus 229E + *Haemophilus* influenza-all types + *M. catarrhalis*
2014113149	Male	3.107	39.6	Yes	Yes	POS	Yes	2+	Yes	*Plasmodium*	Yes	Coronavirus 229E + *Haemophilus* influenza-all types + *S. aureus* + *S. pneumoniae*
2014113150	Male	2.012	39.3	No	Yes	POS	Yes	4+	Yes	None	Yes	*Haemophilus* influenza-all types + *M. catarrhalis* + parainfluenza virus 1 + *S. pneumoniae*
2014113151	Female	10.03	38	No	Yes	POS	Yes	4+	Yes	*Plasmodium*	Yes	Coronavirus 229E + *Haemophilus* influenza-all types + *M. catarrhalis* + *S. aureus*
2014113152	Male	9.382	37.6	No	Yes	POS	Yes	2+	Yes	None	Yes	*Haemophilus* influenza-all types + respiratory syncytial virus + *S. aureus* + *S. pneumoniae*
2014113153	Male	6.201	40.2	Yes	Yes	POS	Yes	1+	Yes	*Plasmodium*	Yes	*Haemophilus* influenza-all types + *M. catarrhalis* + *P. aeruginosa* + *S. pneumoniae*
2014113154	Female	1.333	37.8	No	Yes	POS	Yes	2+	Yes	None	Yes	Group A *Streptococcus* + *Haemophilus* influenza-all types + *M. catarrhalis* + *S. aureus*

(continued)

Supplemental Table 1

Continued

Sample ID	Gender	Age	Axillary temperature (°C)	Admission	Malaria RDT completed?	RDT result	Microscopy completed?	Microscopy result	AFI TAC completed?	Dectection(s) by AFI TAC result	Respiratory TAC completed?	Detection(s) by respiratory TAC
2014113155	Male	7.69	37.6	No	Yes	NEG	No	–	Yes	None	Yes	*Haemophilus* influenza-all types + *Haemophilus* influenza-type B1 + *M. catarrhalis* + *S. pneumoniae*
2014113156	Male	25.07	38.4	No	Yes	POS	Yes	No parasite	Yes	None	Yes	Group A *Streptococcus* + *Haemophilus* influenza-all types + *S. aureus* + *S. pneumoniae*
2014113157	Male	6.329	38	No	Yes	POS	Yes	1+	Yes	*Plasmodium*	Yes	*Haemophilus* influenza-all types + *M. catarrhalis* + human rhinovirus + *S. pneumoniae*
2014113158	Male	23.5	39	Yes	Yes	NEG	Yes	No parasite	Yes	*Plasmodium*	Yes	*Haemophilus* influenza-all types + *K. pneumoniae* + *S. aureus* + *S. pneumoniae*
2014113159	Male	3.874	37.7	No	Yes	POS	Yes	2+	Yes	None	Yes	Adenovirus + *Haemophilus* influenza-all types + *M. catarrhalis* + respiratory syncytial virus
2014113160	Female	33.19	37.5	No	Yes	POS	Yes	1+	Yes	None	Yes	Adenovirus + Hcv2 + *Haemophilus* influenza-all types + *P. aeruginosa*
2014113161	Female	5.995	40.2	Yes	Yes	NEG	Yes	No parasite	Yes	None	Yes	*Haemophilus* influenza-all types + *M. catarrhalis* + *S. aureus* + *S. pneumoniae*
2014113162	Male	10.07	37.5	No	Yes	POS	Yes	3+	Yes	None	Yes	Group A *Streptococcus* + *M. catarrhalis* + human rhinovirus + *S. pneumoniae*
2014113163	Male	29.85	39	Yes	Yes	POS	Yes	No parasite	Yes	*Plasmodium*	Yes	Group A *Streptococcus* + coronavirus 229E + *Haemophilus* influenza-all types + *S. pneumoniae*
2014113164	Male	8.449	38.2	No	Yes	POS	Yes	2+	Yes	*Plasmodium*	Yes	Enterovirus + *Haemophilus* influenza-all types + human rhinovirus + *S. aureus*
2014113168	Male	3.025	39	Yes	Yes	POS	Yes	3+	Yes	None	Yes	Enterovirus + *M. catarrhalis* + respiratory syncytial virus + *S. pneumoniae*
2014113169	Male	1.697	38.6	No	Yes	NEG	Yes	No parasite	No	None	Yes	Adenovirus + *Haemophilus* influenza-all types + *M. catarrhalis* + *S. pneumoniae*
2014113171	Male	20.65	38.6	No	Yes	POS	Yes	No parasite	Yes	None	Yes	Adenovirus + group A *Streptococcus* + *Haemophilus* influenza-all types + *S. aureus*
2014113172	Male	30.96	39.6	Yes	Yes	POS	Yes	No parasite	Yes	None	Yes	*Haemophilus* influenza-all types + *Haemophilus* influenza-type B2 + *M. catarrhalis* + *S. pneumoniae*
2014113173	Female	2.647	37.7	Yes	Yes	NEG	No	–	No	None	Yes	Adenovirus + *Haemophilus* influenza-all types + *M. catarrhalis* + *S. aureus*
2014113174	Female	7.917	38	Yes	Yes	NEG	Yes	No parasite	Yes	None	Yes	Adenovirus + group A *Streptococcus* + *Haemophilus* influenza-all types + *M. catarrhalis* + *S. pneumoniae*
2014113176	Male	21.44	38.8	Yes	Yes	NEG	No	–	Yes	*Plasmodium*	Yes	Coronavirus 229E + *Haemophilus* influenza-all types + *Haemophilus* influenza-type B1 + respiratory syncytial virus + *S. pneumoniae*
2014113177	Female	1.995	37.5	No	Yes	NEG	No	–	Yes	None	Yes	Adenovirus + *Haemophilus* influenza-all types + *M. catarrhalis* + *P. aeruginosa* + *S. pneumoniae*

(continued)

Supplemental Table 1

Continued

Sample ID	Gender	Age	Axillary temperature (°C)	Admission	Malaria RDT completed?	RDT result	Microscopy completed?	Microscopy result	AFI TAC completed?	Dectection(s) by AFI TAC result	Respiratory TAC completed?	Detection(s) by respiratory TAC
2014113191	Female	4.26	38.6	No	Yes	POS	Yes	No parasite	Yes	*Plasmodium*	Yes	Influenza B + *Haemophilus* influenza-all types + *Haemophilus* influenza-type B1 + *M. catarrhalis* + *S. pneumoniae*
2014113193	Male	2.743	37.8	Yes	Yes	NEG	Yes	No parasite	Yes	*Plasmodium*	Yes	Group A *Streptococcus* + *Haemophilus* influenza-all types + *M. catarrhalis* + respiratory syncytial virus + *S. pneumoniae*
2014113195	Male	6.746	39.1	Yes	Yes	POS	Yes	No parasite	Yes	*Plasmodium*	Yes	Adenovirus + *Haemophilus* influenza-all types + *M. catarrhalis* + human rhinovirus + *S. pneumoniae*
2014113206	Female	4.235	38	Yes	Yes	POS	Yes	4+	Yes	*Plasmodium*	Yes	Enterovirus + group A *Streptococcus* + *Haemophilus* influenza-all types + *M. catarrhalis* + *S. pneumoniae*
2014113207	Female	6.913	38.5	Yes	Yes	POS	Yes	2+	Yes	*Plasmodium*	Yes	Adenovirus + group A *Streptococcus* + *Haemophilus* influenza-all types + *M. catarrhalis* + *S. pneumoniae*
2014113208	Female	5.037	39.2	Yes	Yes	NEG	No	–	Yes	*Plasmodium*	Yes	Group A *Streptococcus* + *Haemophilus* influenza-all types + *M. catarrhalis* + human rhinovirus + *S. pneumoniae*
2014113209	Female	3.542	38.2	Yes	Yes	POS	Yes	3+	Yes	*Plasmodium*	Yes	*Haemophilus* influenza-all types + *M. catarrhalis* + respiratory syncytial virus + human rhinovirus + *S. pneumoniae*
2014113210	Male	3.509	37.6	No	Yes	POS	Yes	3+	Yes	None	Yes	Enterovirus + *Haemophilus* influenza-all types + *M. catarrhalis* + *S. aureus* + *S. pneumoniae*
2014113211	Male	2.573	37.9	Yes	Yes	POS	Yes	3+	No	None	Yes	Group A *Streptococcus* + *Haemophilus* influenza-all types + *M. catarrhalis* + human rhinovirus + *S. pneumoniae*
20141129622	Female	17.6	38.3	Yes	Yes	POS	Yes	3+	Yes	None	Yes	Adenovirus + *Haemophilus* influenza-all types + *M. catarrhalis* + *S. aureus* + *S. pneumoniae*
20141129652	Male	13.67	37.6	No	Yes	POS	Yes	3+	Yes	*Plasmodium*	Yes	Coronavirus 229E + *Haemophilus* influenza-all types + *Haemophilus* influenza-type B1 + *M. catarrhalis* + *S. pneumoniae*
20141129662	Male	13.36	37.6	No	Yes	POS	Yes	3+	Yes	None	Yes	Enterovirus + *Haemophilus* influenza-all types + *M. catarrhalis* + *P. aeruginosa* + human rhinovirus + *S. aureus* + *S. pneumoniae*
2014112960	Female	19.16	38.4	Yes	Yes	NEG	No	–	Yes	*Plasmodium*	No	–
2014112961	Male	21.52	38.3	No	Yes	NEG	No	–	Yes	*Plasmodium*	No	–
2014112962	Male	43.46	37.5	No	Yes	POS	Yes	1+	Yes	*Plasmodium*	No	–
2014112963	Male	22.3	38.4	Yes	Yes	NEG	No	–	Yes	*Plasmodium*	No	–
2014112965	Male	28.12	37.7	No	Yes	POS	Yes	2+	Yes	*Plasmodium*	No	–
2014112966	Male	5.861	38.3	Yes	Yes	NEG	Yes	No parasite	Yes	*Plasmodium*	No	–
2014112967	Male	32.47	39.2	No	Yes	POS	Yes	2+	Yes	None	No	–
2014112970	Female	60	37.7	No	Yes	POS	Yes	4+	Yes	None	No	–
2014112973	Female	11.45	39.6	Yes	Yes	NEG	No	–	Yes	*Plasmodium*	No	–
2014112974	Female	23	38.2	Yes	Yes	POS	Yes	2+	Yes	None	No	–

(continued)

Supplemental Table 1
Continued

Sample ID	Gender	Age	Axillary temperature (°C)	Admission	Malaria RDT completed?	RDT result	Microscopy completed?	Microscopy result	AFI TAC completed?	Dectection(s) by AFI TAC result	Respiratory TAC completed?	Detection(s) by respiratory TAC
2014112977	Male	21	38	Yes	Yes	NEG	No	–	Yes	*Plasmodium*	No	–
2014112979	Male	39.47	39	No	Yes	NEG	No	–	Yes	None	No	–
2014112983	Male	19.7	38.1	Yes	Yes	NEG	No	–	Yes	None	No	–
2014112984	Male	20.07	38.5	No	Yes	NEG	No	–	Yes	None	No	–
2014112985	Male	27.69	39	Yes	Yes	POS	Yes	4+	Yes	*Plasmodium*	No	–
2014112986	Male	20.87	39	Yes	Yes	NEG	No	–	Yes	None	No	–
2014112987	Female	33.28	37.5	No	Yes	POS	Yes	4+	Yes	None	No	–
2014112989	Male	29.76	39.5	Yes	Yes	NEG	No	–	Yes	*Plasmodium*	No	–
2014112990	Male	41.24	38	Yes	Yes	POS	Yes	1+	Yes	*Plasmodium*	No	–
2014112991	Male	36.61	38.1	Yes	Yes	POS	Yes	2+	Yes	None	No	–
2014112992	Female	1.99	39.5	Yes	Yes	POS	Yes	3+	No	None	No	–
2014112993	Female	8.314	37.7	Yes	Yes	POS	Yes	No parasite	No	None	No	–
2014112994	Male	23.94	38.6	No	Yes	NEG	No	–	Yes	*Plasmodium*	No	–
2014112995	Male	54.44	39	No	Yes	NEG	No	–	Yes	*Plasmodium*	No	–
2014112996	Female	23	37.5	No	Yes	POS	Yes	2+	Yes	*Plasmodium*	No	–
2014112999	Male	26.24	38.6	No	Yes	POS	No	–	Yes	*Plasmodium*	No	–
2014113000	Female	9.01	38.5	Yes	Yes	POS	Yes	4+	Yes	*Plasmodium*	No	–
2014113001	Female	33.3	38.5	No	Yes	NEG	No	–	No	None	No	–
2014113002	Male	23.92	38.9	No	Yes	NEG	No	–	Yes	None	No	–
2014113005	Male	40.23	40.2	Yes	Yes	POS	Yes	3+	Yes	*Plasmodium*	No	–
2014113007	Male	34.19	38.1	No	Yes	NEG	No	–	Yes	None	No	–
2014113009	Female	51.01	39.9	No	Yes	POS	Yes	3+	Yes	*Plasmodium*	No	–
2014113010	Female	50.04	39.9	Yes	Yes	NEG	No	–	Yes	*Plasmodium*	No	–
2014113011	Female	47.01	37.5	No	Yes	NEG	No	–	Yes	*Plasmodium*	No	–
2014113013	Male	37.59	37.5	No	Yes	POS	Yes	No parasite	Yes	None	No	–
2014113014	Male	39.27	38.2	Yes	Yes	NEG	No	–	Yes	None	No	–
2014113015	Male	21.96	37.8	No	Yes	NEG	No	–	Yes	None	No	–
2014113016	Female	1.251	38.6	No	Yes	NEG	No	–	No	None	No	–
2014113017	Male	46.57	38	No	Yes	POS	Yes	No parasite	Yes	None	No	–
2014113018	Female	8.81	40.4	Yes	Yes	NEG	No	–	Yes	*Plasmodium*	No	–
2014113019	Female	1.3	38.1	No	Yes	NEG	No	–	No	None	No	–
2014113020	Male	32.41	37.9	No	Yes	NEG	No	–	Yes	None	No	–
2014113021	Female	74.03	38.9	Yes	Yes	POS	Yes	No parasite	Yes	None	No	–
2014113022	Female	25.84	37.5	No	Yes	POS	Yes	No parasite	Yes	*Plasmodium*	No	–
2014113023	Female	23.71	38.3	Yes	Yes	POS	Yes	2+	Yes	None	No	–
2014113024	Female	18.92	38.2	No	Yes	POS	Yes	1+	Yes	None	No	–
2014113025	Female	45.75	38	Yes	Yes	POS	Yes	No parasite	Yes	*Plasmodium*	No	–
2014113026	Male	13.51	37.6	Yes	Yes	NEG	No	–	Yes	*Plasmodium*	No	–
2014113027	Female	40.05	39.9	Yes	Yes	POS	Yes	2+	Yes	*Plasmodium*	No	–
2014113028	Male	17.64	40	Yes	Yes	NEG	No	–	Yes	Lepto	No	–
2014113029	Female	4.098	37.8	Yes	Yes	POS	Yes	2+	Yes	*Plasmodium*	No	–
2014113030	Male	34.49	39	Yes	Yes	NEG	No	–	Yes	None	No	–
2014113031	Female	21.82	38	Yes	Yes	POS	No	–	Yes	None	No	–
2014113032	Female	24.44	37.5	No	Yes	POS	Yes	4+	Yes	*Plasmodium*	No	–
2014113033	Female	33.67	39	Yes	Yes	NEG	No	–	Yes	None	No	–
2014113034	Male	12.12	38	Yes	Yes	POS	Yes	4+	Yes	*Plasmodium*	No	–
2014113035	Male	5.07	38.6	Yes	Yes	NEG	No	–	Yes	*Plasmodium*	No	–
2014113036	Male	1.713	38.5	Yes	Yes	POS	Yes	4+	Yes	None	No	–
2014113037	Male	1.147	38.4	Yes	Yes	NEG	No	–	No	None	No	–

(continued)

Supplemental Table 1

Continued

Sample ID	Gender	Age	Axillary temperature (°C)	Admission	Malaria RDT completed?	RDT result	Microscopy completed?	Microscopy result	AFI TAC completed?	Dectection(s) by AFI TAC result	Respiratory TAC completed?	Detection(s) by respiratory TAC
2014113039	Female	24.3	37.6	Yes	Yes	POS	Yes	No parasite	Yes	None	No	–
2014113040	Female	62.04	37.9	Yes	Yes	NEG	No	–	Yes	None	No	–
2014113041	Female	34.96	38.4	No	Yes	POS	No	–	Yes	None	No	–
2014113042	Male	30.05	38.3	No	Yes	POS	Yes	2+	Yes	None	No	–
2014113043	Male	41.8	39	No	Yes	POS	Yes	4+	Yes	*Plasmodium*	No	–
2014113044	Female	33.04	38.1	No	Yes	NEG	No	–	Yes	None	No	–
2014113045	Male	48.04	37.9	Yes	Yes	POS	Yes	2+	Yes	*Plasmodium*	No	–
2014113046	Male	25.48	40	Yes	Yes	NEG	No	–	Yes	*Plasmodium*	No	–
2014113047	Male	30.13	38.8	No	Yes	NEG	No	–	Yes	*Plasmodium*	No	–
2014113048	Male	28.25	38.2	Yes	Yes	NEG	Yes	No parasite	Yes	*Plasmodium*	No	–
2014113049	Male	23.32	40.1	Yes	Yes	POS	Yes	3+	Yes	*Plasmodium*	No	–
2014113050	Male	23.52	38.5	Yes	Yes	NEG	No	–	Yes	*Plasmodium*	No	–
2014113051	Male	29.73	39.3	Yes	Yes	NEG	No	–	Yes	*Plasmodium*	No	–
2014113052	Male	24.21	39	Yes	Yes	NEG	No	–	Yes	*Plasmodium*	No	–
2014113053	Male	23.19	37.5	Yes	Yes	NEG	No	–	Yes	None	No	–
2014113054	Male	20.91	38	Yes	Yes	POS	Yes	No parasite	Yes	None	No	–
2014113055	Male	19.49	38.1	Yes	Yes	POS	Yes	1+	Yes	*Plasmodium*	No	–
2014113056	Male	26.27	38.7	Yes	Yes	POS	Yes	2+	Yes	*Plasmodium*	No	–
2014113059	Male	49.53	37.5	No	Yes	NEG	No	–	Yes	*Plasmodium*	No	–
2014113060	Male	1.815	39.6	Yes	Yes	POS	Yes	1+	Yes	None	No	–
2014113061	Male	6.067	38.1	Yes	Yes	NEG	No	–	Yes	*Plasmodium*	No	–
2014113062	Male	4.38	38.9	Yes	Yes	POS	Yes	2+	Yes	*Plasmodium*	No	–
2014113063	Male	4.796	40	Yes	Yes	POS	Yes	3+	Yes	*Plasmodium*	No	–
2014113064	Male	32.85	39.5	No	Yes	POS	Yes	2+	Yes	*Plasmodium*	No	–
2014113065	Female	48.61	37.6	No	Yes	POS	Yes	2+	Yes	None	No	–
2014113066	Male	34.9	38	No	Yes	POS	Yes	4+	Yes	*Plasmodium*	No	–
2014113067	Male	60.02	38.5	Yes	Yes	NEG	No	–	Yes	*Plasmodium*	No	–
2014113068	Male	57.03	37.5	No	Yes	NEG	No	–	Yes	*Plasmodium*	No	–
2014113070	Female	6.017	38	Yes	Yes	NEG	No	–	No	None	No	–
2014113071	Male	24.23	38	No	Yes	POS	Yes	2+	No	None	No	–

AFI = acute febrile illness; TAC = TaqMan array cards.
